# The effect of a community health worker intervention on public satisfaction: evidence from an unregistered outcome in a cluster-randomized controlled trial in Dar es Salaam, Tanzania

**DOI:** 10.1186/s12960-019-0355-7

**Published:** 2019-03-29

**Authors:** Elysia Larson, Pascal Geldsetzer, Eric Mboggo, Irene Andrew Lema, David Sando, Anna Mia Ekström, Wafaie Fawzi, Dawn W. Foster, Charles Kilewo, Nan Li, Lameck Machumi, Lucy Magesa, Phares Mujinja, Ester Mungure, Mary Mwanyika-Sando, Helga Naburi, Hellen Siril, Donna Spiegelman, Nzovu Ulenga, Till Bärnighausen

**Affiliations:** 1 Department of Global Health and Population, Harvard T.H. Chan School of Public Health, 665 Huntington Ave., Building 1, 11th floor, Boston, MA 02115 United States of America; 2 Management and Development for Health, Dar es Salaam, Tanzania; 3 Department of Public Health Sciences, Karolinska Institutet, Stockholm, Sweden; 4 Department of Psychiatry, Yale School of Medicine, New Haven, United States of America; 5 Muhimbili University of Health and Allied Sciences, Dar es Salaam, Tanzania; 6 Janssen Research & Development, LLC, Raritan, United States of America; 7 Africa Academy for Public Health, Dar es Salaam, Tanzania; 8 Department of Epidemiology, Harvard T.H. Chan School of Public Health, Boston, United States of America; 9 Heidelberg Institute of Global Health, University of Heidelberg, Heidelberg, Germany; 10 Center for Methods on Implementation and Prevention Science and Department if Biostatistics, Yale School of Public Health, New Haven, CT USA

**Keywords:** Community health workers, Satisfaction, Sub-Saharan Africa, Maternal and child health, Task shifting

## Abstract

**Background:**

There is a dearth of evidence on the causal effects of different care delivery approaches on health system satisfaction. A better understanding of public satisfaction with the health system is particularly important within the context of task shifting to community health workers (CHWs). This paper determines the effects of a CHW program focused on maternal health services on public satisfaction with the health system among women who are pregnant or have recently delivered.

**Methods:**

From January 2013 to April 2014, we carried out a cluster-randomized controlled health system implementation trial of a CHW program. Sixty wards in Dar es Salaam, Tanzania, were randomly allocated to either a maternal health CHW program (36 wards) or the standard of care (24 wards). From May to August 2014, we interviewed a random sample of women who were either currently pregnant or had recently delivered a child. We used five-level Likert scales to assess women’s satisfaction with the CHW program and with the public-sector health system in Dar es Salaam.

**Results:**

In total, 2329 women participated in the survey (response rate 90.2%). Households in intervention areas were 2.3 times as likely as households in control areas to have ever received a CHW visit (95% CI 1.8, 3.0). The intervention led to a 16-percentage-point increase in women reporting they were satisfied or very satisfied with the CHW program (95% CI 3, 30) and a 15-percentage-point increase in satisfaction with the public-sector health system (95% CI 3, 27).

**Conclusions:**

A CHW program for maternal and child health in Tanzania achieved better public satisfaction than the standard CHW program. Policy-makers and implementers who are involved in designing and organizing CHW programs should consider the potential positive impact of the program on public satisfaction.

**Trial registration:**

ClinicalTrials.gov, EJF22802

## Background

Public satisfaction with the health system is important for several reasons. First, it is a key goal of health systems in and of itself. For instance, the World Health Organization (WHO) considers public satisfaction with the health system, encapsulated in the concept of “health system responsiveness,” to be one of three fundamental objectives of a health system [1]. Healthcare for pregnancy and delivery is a crucial opportunity for countries to affect public satisfaction with their health system given the large proportion of the population that will, either directly or indirectly, be in contact with the health system for pregnancy and childbirth in their lifetime. Second, public satisfaction with the health system is important for instrumental reasons, because poor satisfaction with the health system can result in low uptake of needed healthcare [2]. In the case of health programs for women of reproductive age, high satisfaction with the public-sector health system can contribute to ensuring good coverage of antenatal care, facility-based delivery, and postnatal care, as well as good adherence to nutrition recommendations provided by health workers. For instance, women who are dissatisfied with their maternal care during pregnancy may be less likely to come in for postnatal checkups. Third, public satisfaction with the health system is a key metric to hold actors in the health system—policy-makers, planners, and providers—accountable for their decisions and actions [3].

Health services research usually studies satisfaction with healthcare among those who have attended care (“patient satisfaction”). Even some population-based surveys restrict their questions on healthcare satisfaction to those who report to have recently visited a healthcare facility [4]. From a health system perspective, however, satisfaction with the health system among the entire target population, in this case all women who are pregnant or have recently delivered (henceforth referred to as ‘public satisfaction’), is a more important measure, as it includes those who are not using care. It is precisely these intended beneficiaries of healthcare who are not engaging with the health system who should be the target of interventions to improve public satisfaction with the health system—one of the many reasons they are not using the system could be that they are dissatisfied with the current system [5, 6]. Non-users are also likely to derive a benefit from increased use of healthcare.

**Fig. 1:**
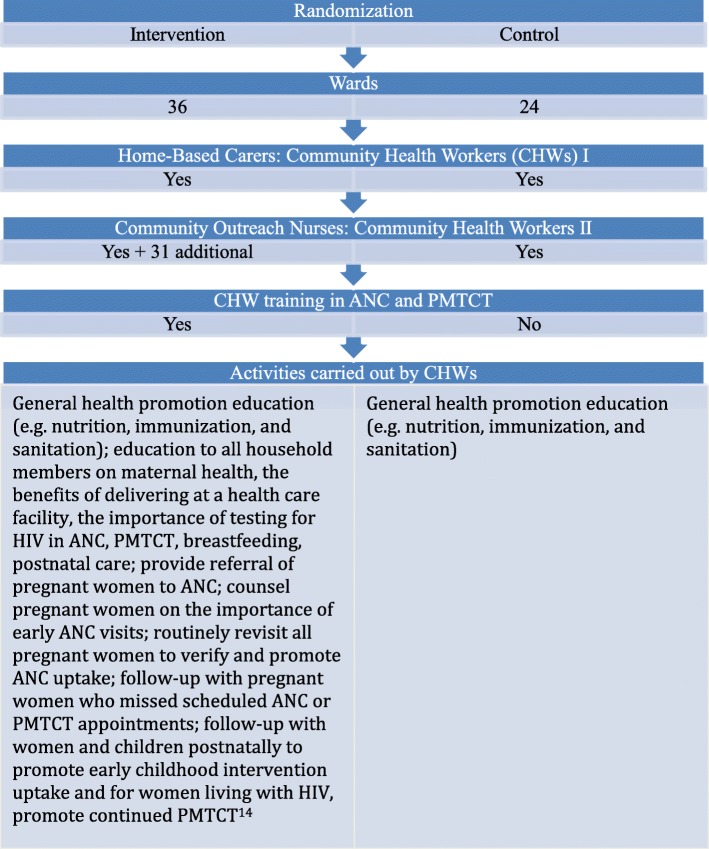
The structure of *Familia Salama*, a community health worker intervention in Dar es Salaam, Tanzania

There is a dearth of evidence on the causal effects of different care delivery approaches on public satisfaction with the health system. Community health worker (CHW) programs are an important instrument to overcome the severe shortage of nurses and physicians in sub-Saharan Africa (SSA) and increase use of healthcare [7]. Many CHW programs are currently being scaled up in countries throughout SSA [8]. In particular, CHWs have been widely used to improve maternal and child health [9–12]. Given their increased use throughout SSA, it is important to understand their effect on public satisfaction both with the CHW program and with the health system. We sought to fill this gap in knowledge by analyzing data from a population-based cluster-randomized controlled trial to measure the effect of a CHW intervention for maternal and child health on public satisfaction with the health system among women who were pregnant or had recently delivered a child. This evaluation compared a CHW intervention that included additional CHWs and additional training (as outlined in Fig. 1) to an existing model of CHW that did not include training specific to maternal health.

## Methods

### Study setting

This population-based cluster-randomized controlled trial took place throughout two out of three districts of Dar es Salaam, Tanzania. Sixty wards in the Kinondoni and Ilala districts of Dar es Salaam were randomly assigned to the intervention and control arms. Because the size of the populations in each ward varied and the goal was to balance the population size in the intervention and control arms, block randomization was used and 36 wards were randomized to the intervention arm and 24 to the control arm (Fig. 1). Six wards in the control arm did not have facilities and were thus not included in data collection. Randomization details have been previously reported [13].

### Exposure: the community health worker intervention

The community health worker intervention has been previously described in detail [13, 14]. In brief, the community health worker (CHW) intervention included two existing cadres of community outreach workers (CHWs or “home-based carers,” and facility-based community outreach nurses). These cadres carried out activities to encourage antenatal care (ANC) and prevention of mother-to-child transmission of HIV (PMTCT) uptake and retention, as well as to counsel women of reproductive age on maternal and child health, nutrition, and hygiene. Each neighborhood was assigned one to three CHWs, and the community outreach nurses supervised these CHWs. Because this intervention built upon the existing CHW structure, there were CHWs in both the intervention and control arms of the study; however, the intervention arm was supplemented with an additional 31 community outreach nurses. In both the intervention and control arms, CHWs carried out general activities to promote healthy behaviors, but the CHW activities in the control arm did not include activities with a specific focus on maternal and child health.

Management and Development for Health (MDH), a Tanzanian-led non-governmental health and development organization, implemented the intervention in partnership with the Tanzanian Ministry of Health and Social Welfare (MOHSW) from January 2013 to April 2014 under the name *Familia Salama* (Swahili for “safe family”). The full research study was a partnership between MDH, MOHSW, and Muhimbili University of Health and Allied Sciences in Tanzania as well as the Harvard T.H. Chan School of Public Health in the United States of America and the Karolinska Institute in Sweden.

### Data collection

We had set out to measure all endpoints of this trial through clinic-based registers. However, because of concerns regarding data quality and problems with linkage across clinical registers, the study team decided while the trial was ongoing to instead conduct a population-based survey at the end of the study period. The results reported here are from this population-based survey. We conducted a population-representative household survey from May to August 2014. The survey followed a two-stage random cluster sampling design. In the first stage, 183 neighborhoods in the intervention and control wards were randomly selected. The neighborhoods are the smallest unit of local government in urban areas in Tanzania [15]. Within each neighborhood, the fieldworkers used a random number generator to select a household. Following the first visit to the household, the fieldworkers visited every fifth household in a randomly selected direction from the first household—until they had visited a total of 60 households in the neighborhood or until they had visited all of the households in this neighborhood. CHWs who had undergone training in data collection methods conducted enumeration and interviews. CHWs did not collect data from the same neighborhood where they worked as CHWs. Women were eligible for inclusion in the household survey if they were either currently pregnant, or if they had delivered a child within the previous 2 years (from June 2012 to May 2014), i.e., during any time within the study intervention period.

This study was approved by the ethics review boards at the National Institutes of Medical Research in Tanzania and at the Harvard T.H. Chan School of Public Health in the United States. The ethics review boards exempted the study from written informed consent for the CHW component of the study; oral informed consent was obtained.

### Variables and analysis

We first assessed the reach of the CHW intervention, using women’s reports of CHW visits. The women were then asked to assess their satisfaction with the public healthcare system in Dar es Salaam on a five-level Likert scale from “very dissatisfied” to “very satisfied.” In addition, respondents were asked to state their satisfaction with the CHW program on the same scale. The survey question did not specify CHWs trained specifically by the intervention. It is thus likely that respondents were evaluating the CHW program they were exposed to, which would have been the standard program in control areas and the enhanced program in intervention areas. The satisfaction outcome variables were not pre-registered primary or secondary endpoints of this trial.

In order to compare each satisfaction level by intervention status, we first used an ordinal logistic regression model. The model provides the odds of a one-level increase in satisfaction in the intervention group compared to the control group. We assessed the proportional odds assumption and found that it was violated for the outcome “satisfied with the CHW program.” This indicates that the change from each level of satisfaction to the next in the ordered scale is not uniform. For our final models, we therefore used linear regression to assess the linear probability of risk of satisfaction at each potential cutoff (e.g., very satisfied versus not very satisfied).

In addition to the intent-to-treat analysis described above, we conducted two sensitivity analyses. First, we used the randomized treatment as an instrument to conduct an instrumental variable (IV) analysis. IV analyses within the context of randomized control trials provide a local average treatment effect, that is, the effect of the treatment among those who would uptake the treatment [16]. We used two-stage least squares to fit a linear probability model for each level of satisfaction on the CHW program. Second, we assessed the potential influence of missing outcome data by imputing the missing values using conditional multiple imputation [17]. The woman’s location, intervention status, age, and interviewer were used as predictors. The MICE package in Stata was used for multiple imputation.

All standard errors are robust, clustered at the ward level. Data analyses were conducted in Stata version 14.1 (StataCorp LP, College Station, United States of America).

## Results

There were 2329 women who participated in this survey (response rate 90%). Women were on average 27.3 years old (range 15–48), and 33% of women had some secondary school education or higher. The demographic characteristics did not differ between intervention and control clusters (Table 1).

**Table 1:** Demographic characteristics of 2 239 women, Dar es Salaam, Tanzania, 2014

Characteristic	Intervention (*n* = 1 664)	Control (*n* = 665)
Age, mean (SD)	27.3 (5.9)	27.4 (5.9)
Education: highest level attended		
Less than primary	103 (6.6%)	55 (8.9%)
Primary	941 (60.2%)	363 (58.7%)
Post-primary	518 (33.2%)	200 (32.4%)
Number of household members, mean (SD)		
≥ 18 years	2.8 (1.1)	2.7 (1.1)
< 18 years	2.0 (1.4)	2.1 (1.5)

Differences in the means and proportions between the study arms are not statistically significant; categories do not sum to the total due to missing data.

*SD* standard deviation

**Fig. 2:**
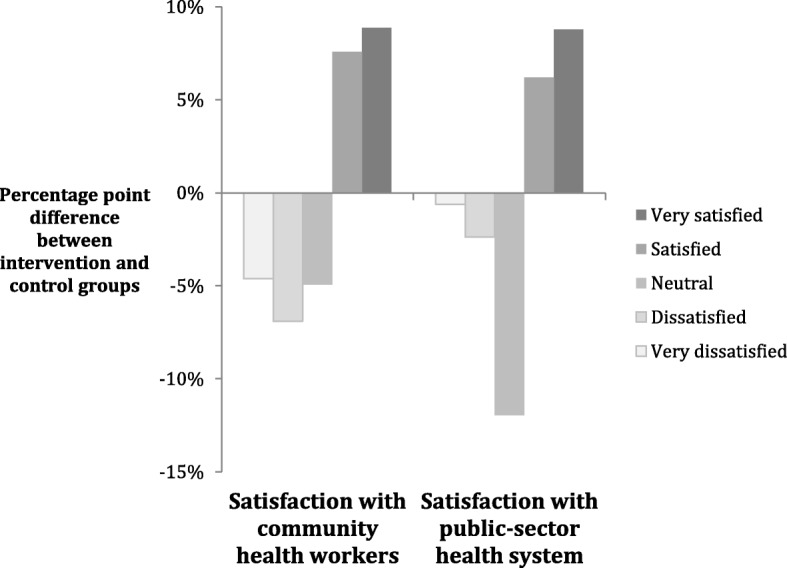
Differences between intervention and control groups’ satisfaction with community health workers and the health system

Of women living in intervention areas, 86% reported that their household had ever received a visit from a CHW, compared to 37% of women living in control areas. Households in intervention areas were thus 2.3 times as likely to have ever received a CHW visit compared to households in control areas (*p* < 0.001). Women in intervention areas reported higher satisfaction with the CHW program and higher satisfaction with the public health system in Dar es Salaam than women in the control areas (Fig. 2).

The ordinal logistic regression estimated a strong positive association between the CHW intervention and satisfaction with both CHWs (*β* = 0.94, *p* = 0.001) and the public-sector health system (*β* = 0.61, *p* = 0.004). For both outcomes of interest, the difference in satisfaction between intervention and control arms was at least borderline significant at each cutoff, e.g., very satisfied, satisfied, neutral, and dissatisfied (Table 2). The proportion of women reporting they were satisfied or very satisfied (rather than neutral, dissatisfied, or very dissatisfied) with the CHW program was 16 percentage points higher (95% CI 3, 30) in the intervention arm than in the control arm, and the proportion of women reporting they were satisfied or very satisfied with the public-sector health system was a significant 15 percentage points higher (95% CI 3, 27) in the intervention arm than in the control arm.

**Table 2:** Effect of community health worker intervention on public satisfaction

	ITT			CACE			ITT with MI		
	Δppt	95% CI	*p* value	Δppt	95% CI	*p* value	Δppt	95% CI	*p* value
Satisfaction with community health worker program									
Very satisfied	9	− 2, 20	0.10 8	18	− 5, 41	0.12 6	10	0, 20	0.04 4
Satisfied or very satisfied	16	3, 30	0.01 6	34	8, 60	0.01 1	18	6, 29	0.00 5
Neutral or above	12	2, 21	0.02 1	25	5, 46	0.01 5	11	3, 19	0.00 8
Dissatisfied or above	5	0, 10	0.06 8	10	0, 21	0.06 0	5	0, 9	0.03 8
Very dissatisfied	0	–	–	0	–	–	0	–	–
*N*	2 077			1 799			2 312		
Satisfaction with the public healthcare system in Dar es Salaam									
Very satisfied	9	0, 17	0.04 8	19	−1, 39	0.06 0	8	0, 17	0.05 8
Satisfied or very satisfied	15	3, 27	0.01 5	35	10, 60	0.00 6	14	3, 26	0.01 8
Neutral or above	3	−5, 11	0.45 6	9	−9, 27	0.34 7	4	−4, 11	0.35 3
Dissatisfied or above	1	−3, 4	0.74 4	1	−6, 9	0.75 2	1	−3, 5	0.64 4
Very dissatisfied	0	–	–	0	–	–	0	–	–
*N*	2 171			1 871			2 312		

ITT—intent-to-treat analysis comparing satisfaction among individuals in the intervention arm to individuals in the control arm using complete case analysis. CACE—sensitivity analysis using two-stage least squares to adjust the effect of the intervention for treatment compliance. The intervention assignment is used as an instrument for receipt of the HBC intervention. ITT with MI—sensitivity analysis using intent-to-treat with missing data multiply imputed using respondents’ location, age, education, and interviewer. All confidence intervals and *p*-values are adjusted for clustering at the ward level. For each level, the point estimate compares the likelihood of giving that response or a more positive response. For example, for the “satisfied” row in “how satisfied are you with the CHW program,” the intervention led to a 16-percentage-point increase in reporting they were satisfied or very satisfied with the CHW program rather than neutral, dissatisfied, or very dissatisfied

*Δppt* percentage point change, *CI* confidence interval, *ITT* intent-to-treat, *CACE* complier average causal effect, *MI* multiple imputation

The results from both sensitivity analyses were similar to the intent-to-treat analysis (Table 2). Outcome data for satisfaction with the HBC program were missing for 252 women (10.8%) and outcome data for satisfaction with the health system in Dar es Salaam were missing for 158 women (6.8%). When we imputed these missing data, we found that the association between intervention and satisfaction with the health system became significant for all but two of the cutoffs.

## Discussion

This cluster-randomized controlled trial in Dar es Salaam provides evidence that adding maternal health support to a CHW program improves women’s satisfaction with both the CHW program itself and the overall public-sector health system. This study is the first to identify a link between CHW programs and public satisfaction with the health system. Public satisfaction with the health system is important for health policy, because it is a key outcome of health system activities, affects the uptake of needed healthcare, and can hold policy-makers and providers accountable for their decisions and actions. While we hope that our findings will stimulate policy-makers to consider CHW programs to improve both maternal health and satisfaction with the health system for intrinsic reasons, the importance of satisfaction with the health system for politicians’ approval ratings indicates that CHW programs can also be of use as instruments of *Realpolitik*. This latter use of health system reforms and interventions to increase political standing and power has a tradition of political actions driven by power considerations that do not rule out population benefits [18].

While the public satisfaction effects of CHW programs have not been studied before, even patient satisfaction has rarely been assessed in CHW studies. In a recent review of 26 studies that assessed the effects of community-based intervention packages for improving maternal and newborn health, Lassi and Bhutta identified only one that had assessed patient satisfaction as an outcome [11]. In the identified study by Magoma and colleagues, the intervention did not greatly affect patient satisfaction among women utilizing antenatal care [19].

There are multiple pathways through which the CHW program in this study may have improved public satisfaction with the health system. First, because the CHWs provided education, women may have felt that they were receiving direct services from the public-sector health system that they had not previously received, therefore increasing their satisfaction with both the CHW program and the overall health system. Second, because the CHWs were able to meet with many women before their first antenatal care visit, CHWs may have been able to help women to better prepare for and navigate the system, leading to better experiences with the public system and improved satisfaction. Third, CHWs may have encouraged more interactions with the health system, leading to higher satisfaction. Fourth, if CHWs are seen as a part of the public system and they provided good interpersonal care, they may have helped to improve individuals’ trust in the health system [20]. Current evidence from Tanzania indicates that disrespectful care is distressingly common during labor and delivery [21, 22] and this disrespectful care leads to reduced satisfaction with, and trust in, the public-sector health system [23]. While the experience of care during and after delivery may be quite different from the experiences with the CHWs, there is evidence of a link between patient experience and satisfaction with other services: dissatisfaction with PMTCT care in the same region was strongly associated with feelings that the provider did not understand the client’s concerns or had poor communication skills [24]. In this context of negative patient experience and disrespectful care, it is possible that the CHWs serve to provide a familiar and personable face to the health system. Satisfaction is a complex construct that incorporates women’s expectations for care together with their experience of care [25]. While the objective of this study was to use a simple measure of satisfaction that could be used by policy-makers in rapid evaluations, future work evaluating the pathways through which CHWs can affect the public’s satisfaction with the health system should explore both expectations and experiences and their relationship to CHWs and changing satisfaction.

While a larger proportion of women in the intervention areas reported having ever received a household visit from a CHW compared to women in the control areas, delivery of the CHW intervention was not ubiquitous, and in this survey, we did not measure when a visit was specific to our intervention. The results from the process evaluation of this intervention found that the CHWs became more successful over time in both reaching women for initial visits prior to their first ANC visit and also following up for a repeat visit [14]. For women who had never been approached by CHWs, it is possible that they were aware of, and affected by, the CHW intervention in their community. An individual’s awareness of the efforts made by the health system may cause increased public satisfaction with the health system, even if they do not experience the full intervention. Alternatively, not receiving a known intervention could lead to decreased public satisfaction with the health system. In a recent qualitative evaluation of non-monetary incentives provided to women for facility deliveries in Uganda, researchers found that when individuals were aware of the incentives, but the incentives were not available to them, there was a negative effect on their perceptions of their delivery experience [26]. In our study, among individuals who did not receive a visit from a CHW, there was no difference in satisfaction with the health system between intervention and control arms, indicating that it is unlikely that the intervention had a strong effect on these individuals one way or another. Implementing a CHW program in an urban setting where many women are already exposed to the healthcare system and also often unavailable during work hours for effective home visits potentially reduced the impact of this intervention and points to the potential of CHW programs to have even stronger effects on public satisfaction with the health system in rural settings.

Our study has several strengths. First, it is population-based, which allows us to measure public satisfaction among the target population of women who are pregnant or have recently delivered a child. This measure includes those individuals who are not using the system for reasons that could be related to their satisfaction, thus giving us a better assessment of the impact of the intervention on the target population. Second, assignment of the intervention was random. This allows us to interpret our results causally. Third, the CHW program was implemented using the National Training Curriculum and thus represents a scalable and contextually relevant intervention. Our study has six main limitations. First, for women who reported that their households had never received a visit from a CHW, it is unclear how these women were rating their satisfaction with the CHW program. They may be using information that they have heard from others, or they may be giving a default response to the satisfaction question in order to appease the interviewer. Future qualitative work could elicit how women respond to these questions. Second, using CHWs as data collectors may have induced social desirability bias; if respondents knew the surveyors were CHWs, they may have reported higher satisfaction, because they felt socially pressured. However, CHWs did not collect data within the areas where they provided services, reducing the likelihood that respondents would know them. Further, the main statistic of interest is the difference between satisfactions in the two groups. It is likely that if social desirability bias played a role, it would have played a role in both the intervention and the control group, thus minimizing the effect on the difference statistic. By using CHWs to collect data, there is also potential for interviewer bias, where the CHWs could have biased respondents, either intentionally or subconsciously, to report more favorably on the CHW program in intervention areas than in control areas. The study team made efforts to prevent this form of bias through training of data collectors and through the use of structured surveys that did not have leading questions. Third, the outcome of interest was missing for a substantial number of women. If women are declining to respond differentially based on their satisfaction with the CHW program or the overall health system, then this could bias our results. However, the results from the sensitivity analyses, including the multiple imputation of missing data, further support our findings. Fourth, we did not evaluate the costs of the CHW program or the quality of care provided by the CHWs. Because CHWs do not replace the work of skilled providers, their additional cost to the system needs to be evaluated against their benefits in future work. The quality of care they provide also must be taken into consideration when evaluating their impact. Fifth, the outcome examined in this analysis was not a pre-registered endpoint of this trial. The results should therefore be interpreted as being hypothesis-generating rather than conclusive evidence. Finally, as with most trials, our results may not be generalizable outside of the study population.

## Conclusions

This study provides evidence that CHW interventions focusing on maternal health can lead to improved public satisfaction with the health system among women who are pregnant or have recently delivered. As policy-makers assess options for organizational changes to healthcare delivery, they should consider the potential for these changes to affect the public’s overall satisfaction with the health system. CHWs may be one effective and efficient way to enhance public satisfaction with the health system. These findings should be applied with attention to the specific health system and population needs and priorities [27].

## Abbreviations

ANC: Antenatal care
CHW: Community health worker
MDH: Management and Development for Health
MOHSW: Tanzanian Ministry of Health and Social Welfare
PMTCT: Prevention of mother-to-child transmission of HIV
SSA: Sub-Saharan Africa
WHO: World Health Organization
